# Identification of four prognostic LncRNAs for survival prediction of patients with hepatocellular carcinoma

**DOI:** 10.7717/peerj.3575

**Published:** 2017-07-18

**Authors:** Zhonghao Wang, Qian Wu, Shu Feng, Yanhua Zhao, Chuanmin Tao

**Affiliations:** 1 Department of Laboratory Medicine, West China Hospital of Sichuan University, Chengdu, China; 2 Microbiology and Metabolic Engineering Key Laboratory of Sichuan Province, School of Life Sciences, Sichuan University, Chengdu, China

**Keywords:** Prognostic, LncRNA, Survival, HCC

## Abstract

**Background:**

As the fifth most common cancer worldwide, Hepatocellular carcinoma (HCC) is also the third most common cause of cancer-related death in China. Several lncRNAs have been demonstrated to be associated with occurrence and prognosis of HCC. However, identification of prognostic lncRNA signature for HCC with expression profiling data has not been conducted yet.

**Methods:**

With the reuse of public available TCGA data, expression profiles of lncRNA for 371 patients with HCC were obtained and analyzed to find the independent prognostic lncRNA. Based on the expression of lncRNA, we developed a risk score model, which was evaluated by survival analysis and ROC (receiver operating characteristic) curve. Enrichment analysis was performed to predict the possible role of the identified lncRNA in HCC prognosis.

**Results:**

Four lncRNAs (*RP11-322E11.5*, *RP11-150O12.3*, *AC093609.1*, *CTC-297N7.9*) were found to be significantly and independently associated with survival of HCC patients. We used these four lncRNAs to construct a risk score model, which exhibited a strong ability to distinguish patients with significantly different prognosis (HR = 2.718, 95% CI [2.103–3.514], *p* = 2.32e−14). Similar results were observed in the subsequent stratification survival analysis for HBV infection status and pathological stage. The ROC curve also implied our risk score as a good indicator for 5-year survival prediction. Furthermore, enrichment analysis revealed that the four signature lncRNAs may be involved in multiple pathways related to tumorigenesis and prognosis.

**Discussion:**

Our study recognized four lncRNAs to be significantly associated with prognosis of liver cancer, and could provide novel insights into the potential mechanisms of HCC progression. Additionally, *CTC-297N7.9* may influence the downstream *TMEM220* gene expression through cis-regualtion. Nevertheless, further well-designed experimental studies are needed to validate our findings.

## Introduction

Hepatocellular carcinoma, accounting for 90% of primary liver cancers, is one of the most common malignancies worldwide particularly in East Asia and sub-Saharan Africa. More than 250,000 new cases and approximately 500,000–600,000 deaths occur due to this disease annually (Forner, Llovet & Bruix, 2012). Despite the recent advances in early diagnosis, imaging techniques and therapeutic intervention for HCC, low overall 5-year survival rate of HCC patients remains unsatisfactory (Villanueva, Hernandez-Gea & Llovet, 2013).

LncRNAs, defined as ncRNAs longer than 200 nucleotides, play essential roles in almost every aspect of biological processes. Over the past decades, emerging evidences have associated the misexpression of lncRNAs with tumor initiation, metastasis, and prognosis (Spizzo et al., 2012; Zeng et al., 2017; Zhou et al., 2016). Many studies, up to now, have highlighted the molecular mechanism and biological characters of lncRNA in HCC occurrence and progression (Quagliata et al., 2014; Yang et al., 2011). Moreover, some lncRNAs can also be served as valuable prognostic predictor for HCC patients (Chen et al., 2016; Li et al., 2016; Zhang et al., 2016).

With the development of high-throughput technologies, such as RNA-seq and microarray, tremendous expression datasets of gene transcripts have been created for multiple cancer types. TCGA (The Cancer Genome Atlas), a project comprised of 33 cancer types, aiming to improve the prevention, diagnosis, and treatment of cancer (Cancer Genome Atlas Research, 2011). In our present study, expression data and related clinical information of 371 LIHC (liver hepatocellular carcinoma) patients were obtained from TCGA, then four lncRNAs were recognized as independent prognostic biomarkers in the survival analysis. In addition, we also investigated the possible biological mechanism uncovering the association of these prognostic lncRNAs with cancer occurrence and progression.

## Materials and Methods

### Collection of TCGA dataset and related clinical information

The RNA-Seq data of patients in TCGA-LIHC project were downloaded from Genomic Data Commons Data Portal, as well as the corresponding clinical information (Cancer Genome Atlas Research, 2011). To obtain lncRNA expression profile, we first got ensembl ID of lncRNAs which had been filtered by The Atlas of Noncoding RNAs in Cancer (TANRIC) (Li et al., 2015), then 12,309 lncRNA ID were used to retrieve the required expression information from above RNA-Seq data. Similarly, 19,817 mRNAs ID were downloaded from GENCODE (release 26) (Harrow et al., 2012), then we got the expression profiles of mRNAs from the RNA-Seq data.

### Identification of potential prognostic signatures

After removing lncRNAs whose expression is zero in more than 50% of the samples, a total of 4,683 lncRNAs were utilized to do the differential expression analysis among normal and LIHC. To find the prognostic lncRNAs, these patients were analyzed by univariate and multivariate Cox proportional hazards regression methods. Briefly, univariate survival analysis was performed with expression value of lncRNAs as independent variable, while multivariate analysis was conducted with expression value of lncRNAs and other factors (including clinical features or other lncRNAs) as the independent variables. To construct the predictive risk score model, we only used those lncRNAs which were still independent of other factors after the multivariate Cox regression analysis. The coefficient of each prognostic lncRNA in risk score model was derived from corresponding multivariate analysis of these identified lncRNA markers (Zhou et al., 2015). All patients were divided into two groups according to the risk score. Then, for mRNAs differentially expressed between these two group, prognostic mRNAs were recognized with the same method of lncRNA, as well as the mRNA-based model. The combined transcripts based model was fitted with both above identified lncRNA and mRNA biomarkers.

### Statistical analysis

All bioinformatics analysis and statistical tests were performed with R language (version 3.3.2): (i) limma package for differential expression analysis; (ii) survival package for univariate and multivariate Cox proportional hazards regression survival analysis; (iii) pROC package for ROC curve and the area under the ROC curves (AUC) analysis; (iv) Gene ontology (GO), Kyoto Encyclopedia of Genes and Genomes (KEGG), Disease Ontology (DO) enrichment analysis were performed with clusterProfiler package (Yu et al., 2012). The co-expression correlation between *CTC-297N7.9* and *TMEM220* were evaluated with Pearson correlation coefficient. The expression profiles of *TMEM220* in normal tissues and cancer cell lines were retrieved from Genotype-Tissue Expression (GTEx) project and Cancer Cell Line Encyclopedia (CCLE) project, respectively (Barretina et al., 2012; Consortium GT, 2013).

### Validation for the expression values of lncRNAs

First, nucleotide sequences of each identified lncRNA were downloaded from GENCODE (release 26) (Harrow et al., 2012). The probe sequences of microarray were retrieved from matching platform annotation files deposited in Gene Expression Omnibus (GEO) database with R package GEOquery. Next, probes were re-mapped to lncRNA sequences using Magic-BLAST tool (version 1.2.0, from NCBI), and probes which uniquely mapped to the sequence with no mismatch were retained to represent the corresponding lncRNA. For each GEO dataset which focused on HCC, we used GEO2R web application supplied by GEO (Barrett et al., 2013) to validate differential lncRNA expression.

## Results

### Differential expression profile and identification of prognostic lncRNAs

**Figure 1:**
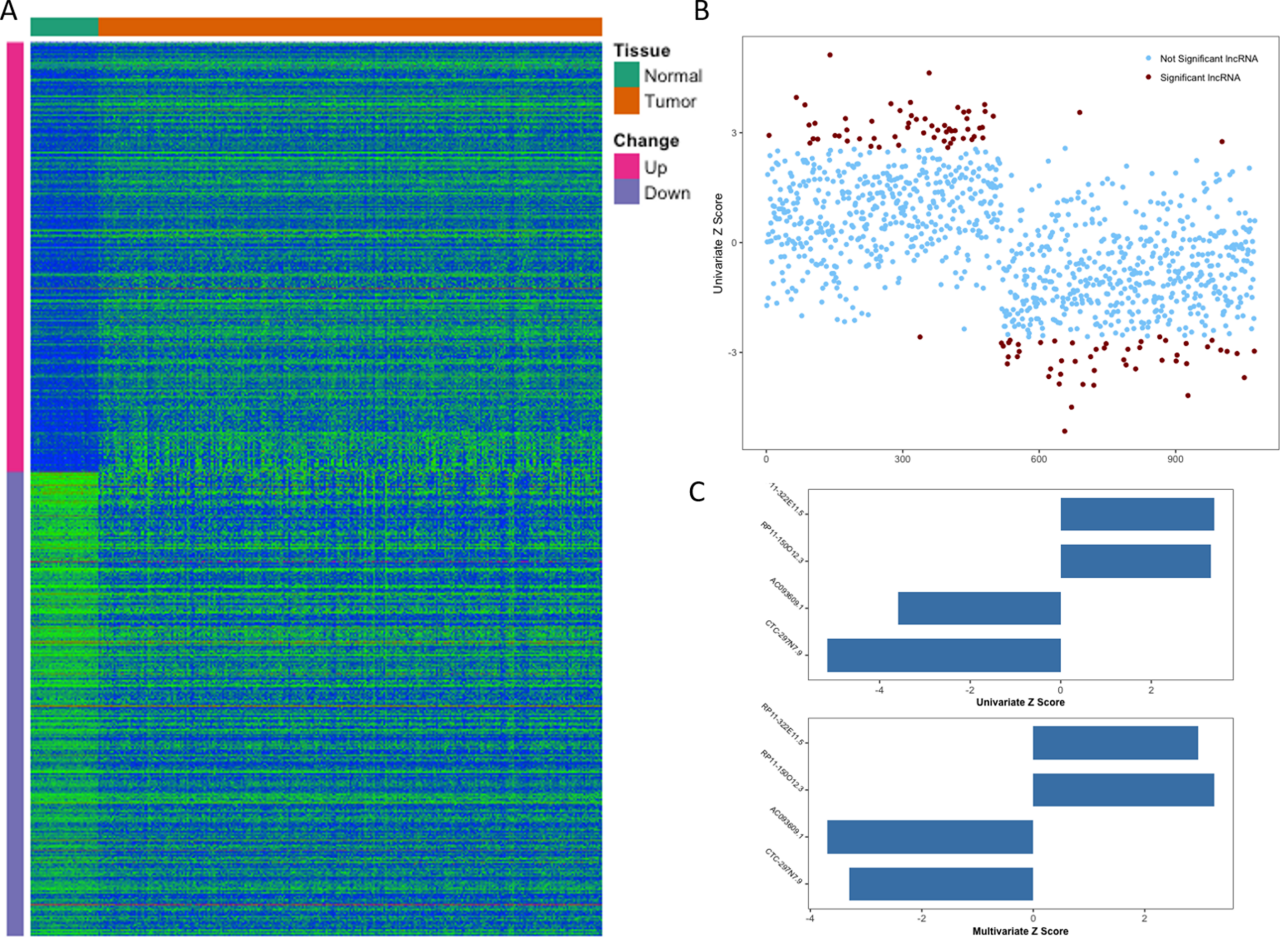
Identification of prognostic lncRNA for patients with HCC. (A) Heatmap of 1,074 differentially expressed lncRNAs between normal and tumor tissues. (B) *Z* score of 1,074 deregulated lncRNAs from univariate Cox aggression analysis (brown dots indicate lncRNAs with *p* value <0.01). (C) *Z* score of four identified prognostic lncRNAs from univariate and multivariate Cox aggression analysis.

From TCGA, RNA-Seq expression profiles of LIHC were downloaded, which covered 371 primary tumor samples and 50 (a subset of 371 HCC patients) paired adjacent normal liver tissue samples. After the initial analysis of all lncRNAs, a total of 1,074 lncRNAs were considered to be differentially expressed between tumor and normal (cutoff of absolute log_2_FC > 1 and adjusted *p*-value <0.01), the expression patterns of which were displayed in Fig. 1A. Then we conducted a univariate Cox proportional hazards regression analysis of the above 1,074 lncRNAs, which revealed a subset of 108 lncRNAs significantly associated with patients’ OS (overall survival, *p*-value <0.01, Fig. 1B). Given that OS can be affected by many factors, a multivariate Cox proportional hazards regression analysis was performed with lncRNAs and other clinical features (such as age of initial diagnosis, pathologic stage and TP53 mutation status) to further evaluate the independent prognostic value of these lncRNAs. Finally, four of the 108 lncRNAs were demonstrated as independent predictors of overall survival (Fig. 1C). The detailed description of these four identified lncRNAs was shown in Table 1.

**Table 1:** Information of the four identified prognostic lncRNAs for HCC.

Ensembl ID	Gene name	Chr.	Coordinate	*Z* score^a^	*P* value^a^
ENSG00000267583	*RP11-322E11.5*	18	35,443,869–35,467,088	3.386	6.935e−04
ENSG00000254290	*RP11-150O12.3*	8	37,597,480–37,599,858	3.313	8.284e−04
ENSG00000230587	*AC093609.1*	2	43,097,746–43,102,691	−3.595	3.143e−04
ENSG00000264016	*CTC-297N7.9*	17	10,741,267–10,769,016	−5.151	2.376e−07

**Notes.**

a Statistics derived from univariate Cox proportional hazards regression analysis.

### Risk score model based on expression level of independent prognostic lncRNAs

**Figure 2:**
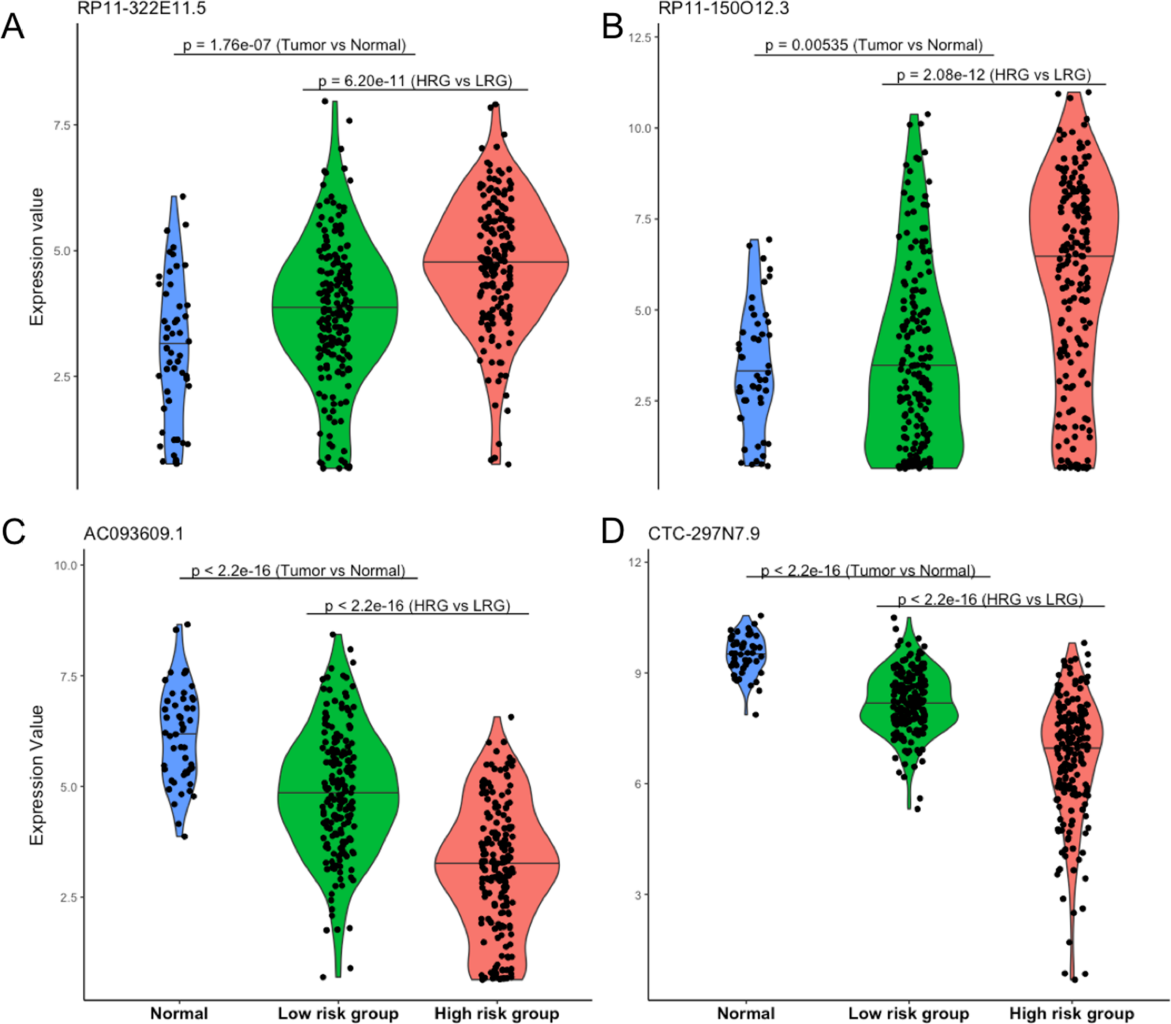
Expression patterns of four prognostic lncRNAs. (A) Expression pattern of *RP11-322E11.5*. (B) Expression pattern of *RP11-150O12.3*. (C) Expression pattern of *AC093609.1*. (D) Expression pattern of *CTC-297N7.9*. Normal indicates paired adjacent tissue from a subset of 371 primary tumors; Low/High risk subgroups were divided by our risk score model; statistical significance between different subgroups were performed with the two-tailed Wilcoxon test.

Four lncRNAs screened from above analysis were used to construct a risk score model, risk score = (0.2567 * expression level of *RP11-322E11.5*) + (0.1307 * expression level of *RP11-150O12.3*) + (−0.2320 * expression level of *AC093609.1*) + (−0.1857 * expression level of *CTC-297N7.9*), then the risk score of each patient was calculated according to this model. With the median value of risk score as cutoff, patients in TCGA datasets were divided into high risk group (HRG, *n* = 186) and low risk group (LRG, *n* = 185). As shown in Fig. 2, the four lncRNAs displayed significantly different expression patterns between normal and tumor, LRG and HRG, which indicated possible protective roles of *AC093609.1* and *CTC-297N7.9*, carcinogenic effects of *RP11-322E11.5* and *RP11-150O12.3* in HCC tumorigenesis and prognosis.

### Performance evaluation of risk score model for survival prediction

**Figure 3:**
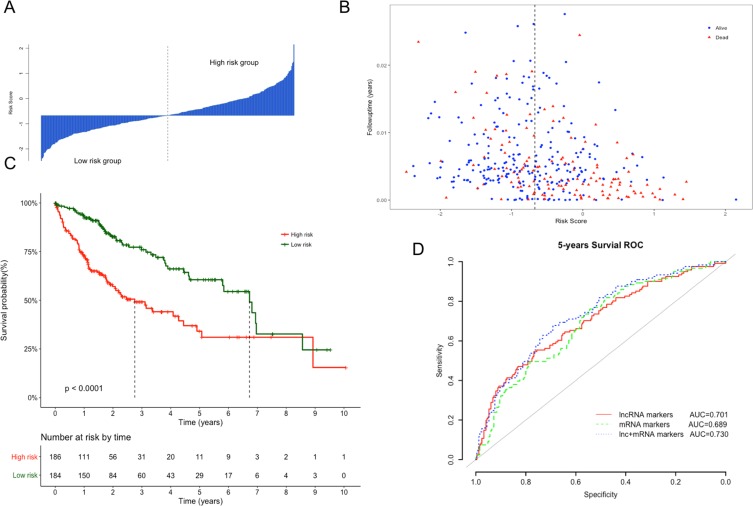
Performance evaluation of risk score model for overall survival prediction. (A) Bar plot of risk score for every patient and ordered by the value of risk score. (B) Distribution of following-up time and risk score for every patient. (C) Kaplan–Meier plots of overall survival for patients with high or low risk score. *P* value was calculated with two-sided log-rank test. (D) ROC curves of the risk score based on prognostic lncRNAs, mRNAs and combined transcripts for five-year survival prediction.

The Kaplan–Meier curves of the above four lncRNAs were presented in Fig. S1. The distribution of risk score, following-up time and survival status were shown in Figs. 3A and 3B. In the survival analysis, K–M plots exhibited a significant difference in patients’ OS between LRG and HRG (log rank *p* < 0.0001, Fig. 3C). Patients in LRG had significantly longer OS (mean 2.64 years) when compared with the HRG (mean 1.76 years). The univariate Cox proportional hazards regression analysis implied the significant association between signature lncRNAs based risk score and patients’ OS (HR = 2.718, 95% CI [2.103–3.514], *p* = 2.32e−14, Table 2). The comparison between prognostic lncRNAs, mRNAs and combined transcripts based risk score model were shown in Table S1.

**Table 2:** Univariate and multivariate Cox proportional hazards regression analysis.

	Univariate analysis		Multivariate analysis	
	Hazard ratio (95% CI)	*P* value	Hazard ratio (95% CI)	*P* value
Risk score^a^	2.718 (2.103–3.514)	2.32E−14	2.513 (1.886–3.348)	3.12E−10
Age	1.01 (0.997–1.024)	0.139	1.009 (0.993–1.025)	0.2602
Gender (Male/Female)	0.816 (0.573–1.163)	0.26	0.968 (0.646–1.450)	0.8748
TP53 mutation (Y/N)	1.563 (1.078–2.266)	0.0184	1.395 (0.915–2.128)	0.1217
HBV infection (Y/N)	1.697 (1.199–2.402)	0.0029	1.656 (1.113–2.463)	0.0129
Pathologic stage (ii/i)	1.423 (0.872–2.323)	0.1583	0.864 (0.513–1.455)	0.5827
Pathologic stage (iii/i)	2.676 (1.754–4.083)	4.92E−06	1.794 (1.125–2.861)	0.0141
Pathologic stage (iv/i)	5.496 (1.695–17.821)	0.0045	2.836 (0.818–9.833)	0.1003

**Notes.**

a Derived from risk score model based on the expression of prognostic lncRNAs.

Furthermore, ROC analysis were also performed to evaluate specificity and sensitivity of the risk score models for five-year survival prediction. The AUC derived from lncRNA risk score model was 0.701, suggesting that our risk score was a good indicator for survival prediction (Fig. 3D). Additionally, risk model developed with the combination of lncRNAs and mRNAs exhibited the best prediction power when compared with only lncRNAs or mRNAs (AUC = 0.73 vs 0.70 or 0.68, respectively). To evaluate the independent predictive performance of our model when considering the traditional clinical prognostic factors, a multivariate Cox regression analysis was conducted with risk score and other clinical features as independent variables in this TCGA dataset. The results revealed a significant correlation with survival time after adjusting for multiple clinical variates (HR = 2.513, 95% CI [1.886–3.348], *p* = 3.12e−10, Table 2).

### Stratified Cox proportional hazards regression analysis

**Figure 4:**
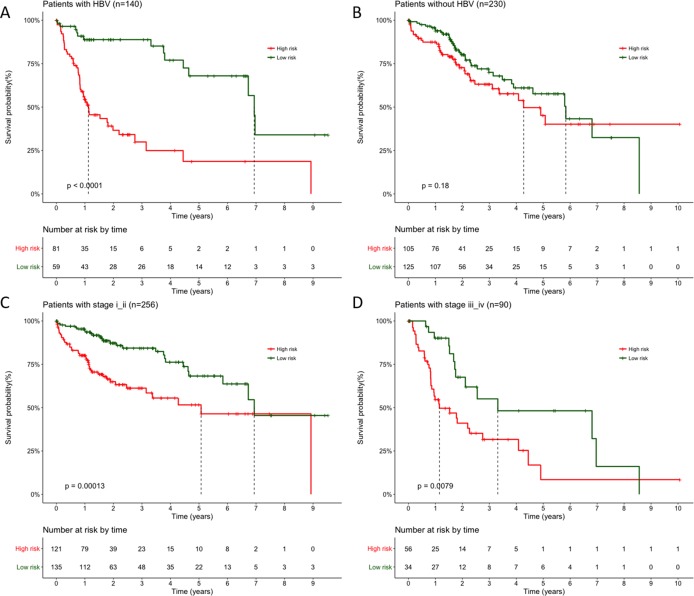
Stratification survival analysis for patients with available HBV infection and pathological information. (A–B) Stratification analysis of patients with or without HBV infection. (C) Stratification analysis of patients with pathological stage i and ii. (D) Stratification analysis of patients with pathological stage iii and iv. *P* value were all calculated with two-sided log-rank test.

Moreover, the results also indicated HBV infection status and pathological stage iii as independent prognostic factors in the above multivariate analysis (*p* = 0.0129 and *p* = 0.0141, respectively), so that we performed stratification analysis for HBV infection status and pathological stage next. All patients were first stratified into group with (*n* = 140) or without HBV infection (*n* = 230). For patients with HBV infection, significant longer OS can still be observed in LRG than HRG (Fig. 4A, *p* < 0.0001, mean 3.04 years vs. 1.31 years), but not for patients without HBV infection (Fig. 4B, *p* = 0.18, mean 2.45 years vs. 2.11 years). Similarly, when 370 patients stratified according to different pathological stage, the Cox regression analysis also exhibited a strong classification power between LRG and HRG both in stage i–ii (Fig. 4C, *n* = 256, *p* = 0.00013, mean 2.75 years vs. 1.88 years) and stage iii–iv subgroup (Fig. 4D, *n* = 90, *p* = 0.0079, mean 2.39 years vs. 1.50 years).

### Expression profiling of identified prognostic lncRNAs

After the examinations of HCC associated datasets in GEO, variations of lncRNA expression were summarized in Table 3. For *AC093609.1*, *CTC-297N7.9* and *RP11-150O12.3*, many other studies manifested the same significant difference as our study in liver tumor tissue (*p* < 0.05). Despite no evidence of significant difference was seen for RP11-322E11.5 (*p* > 0.05), the expression was also up-regulated in GSE98269, which was the same as our result (lgFC > 0).

**Table 3:** Expression profiles of 4 lncRNAs in GEO datasets.

LncRNA	Year	Accession	Normal	Tumor	lgFC	*P* value
RP11-322E11.5	2017	GSE98269	3	3	0.41	4.50E–01
RP11-150O12.3	2008	GSE6222	2	10	1.41	1.60E–01
	2013	GSE50579	10	67	1.93	6.05E–03
	2014	GSE55092	91	49	0.28	3.78E–06
	2014	GSE58043	7	7	1.29	7.16E–02
	2015	GSE67260	5	5	3.17	3.09E–03
	2016	GSE75271	5	50	0.47	2.30E–02
AC093609.1	2008	GSE6222	2	10	−1.88	4.15E–02
	2011	GSE29721	10	10	−0.49	1.33E–03
	2013	GSE17548	20	17	−0.33	4.69E–04
	2013	GSE47197	63	61	−0.6	1.22E–12
	2013	GSE50579	10	67	−1.17	5.68E–05
	2014	GSE62232	10	81	−0.48	2.74E–06
	2014	GSE55092	91	49	−0.54	4.79E–20
	2014	GSE58043	7	7	−1.48	4.48E–02
	2015	GSE67260	5	5	−2.06	6.63E–03
	2017	GSE70880	16	16	−1.48	2.71E–06
	2017	GSE98269	3	3	−1.72	1.11E–02
CTC-297N7.9	2017	GSE98269	3	3	−1.4	2.48E−02
	2015	GSE67260	5	5	−2.24	4.44E–04

### Functional implication of prognostic lncRNA signatures

**Figure 5:**
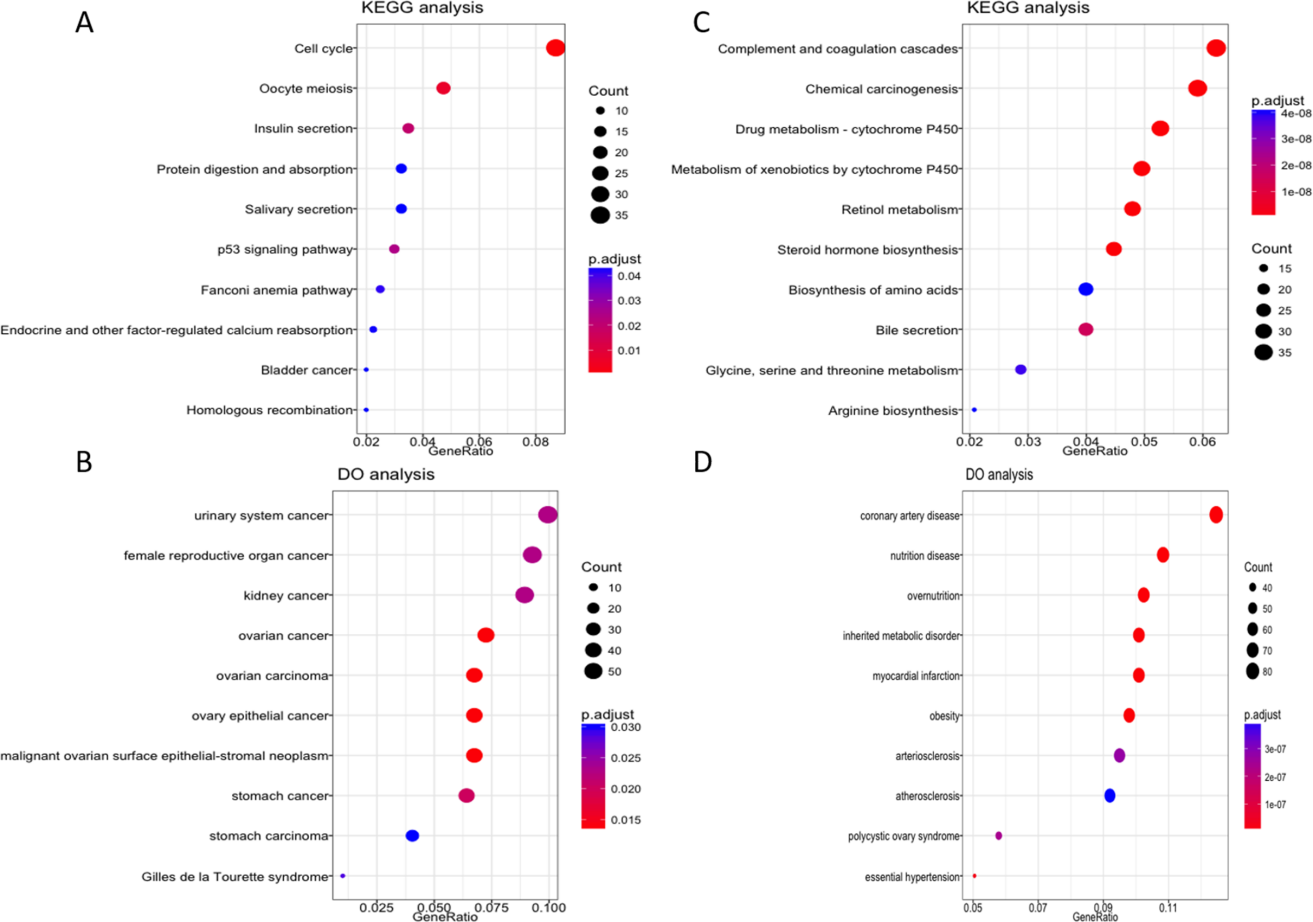
KEGG and DO enrichment analysis of mRNAs deregulated in high risk group compared with low risk group. (A–B) KEGG and DO analysis of up-regulated mRNAs. (C–D) KEGG and DO analysis of down-regulated mRNAs.

To further explore the possible role of these four lncRNA in HCC tumorigenesis and prognosis, we performed enrichment analysis with GO, KEGG and DO databases. First we carried out the expression analysis of mRNA between LRG and HRG, 2,510 in total were recognized as differentially expressed genes. Then we conducted functional enrichment analysis for up-regulated and down-regulated mRNA respectively. The GO, KEGG and DO enrichment showed that 1,189 up-regulated mRNAs are mainly involved in chromosome function, cell cycle, p53 signal pathway and associated with a variety of cancers (Figs. 5A–5B, Fig. S2A). For 1,321 down-regulated mRNAs, it revealed that these mRNAs are participant in many pathways or diseases (Figs. 5C–5D, Fig. S2B), such as blood microparticle, biosynthesis of basic nutrients, drug metabolism and multiple metabolic diseases.

### Biological significance of the prognostic lncRNA *CTC-297N7.9*

**Figure 6:**
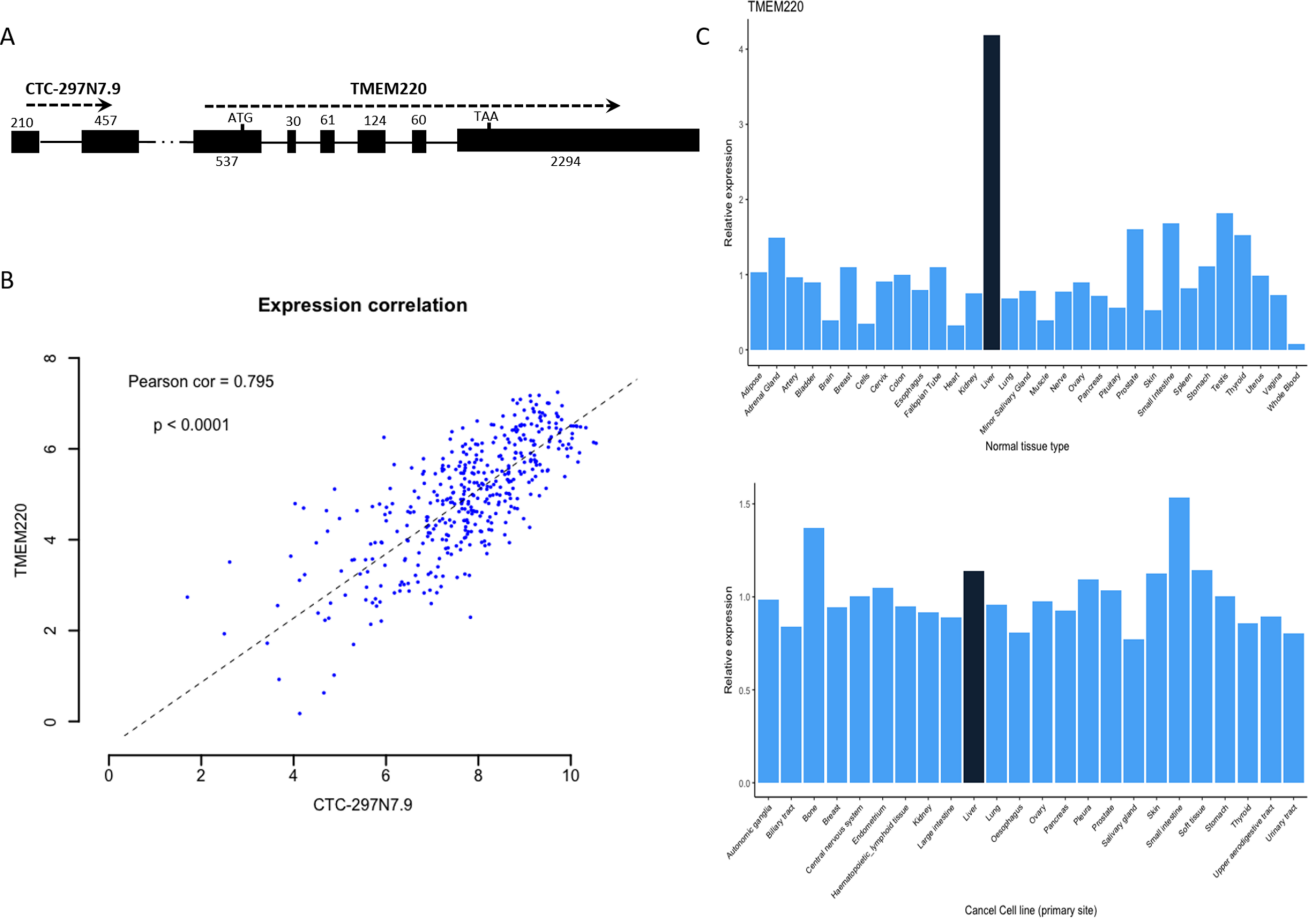
Biological features of prognostic lncRNA *CTC-297N7.9*. (A) Relative genomic location of *CTC-297N7.9* and *TMEM220*. (B) Expression correlation analysis of *CTC-297N7.9* and *TMEM220* in patients with HCC. (C) Expression patterns of *TMEM220* in normal tissues and cancer cell lines. Relative expression was calculated with average expression level of all normal tissues or cancer cell lines as one, respectively.

We further examined whether there were any protein-coding genes which are neighborhoods of these lncRNAs in genomic location coordinates. Among these four prognostic lncRNAs, *CTC-297N7.9* locates upstream of *TMEM220*, which is a transmembrane protein-coding gene (Fig. 6A). Then expression correlation of *CTC-297N7.9* with *TMEM220* were evaluated by Pearson correlation analysis, which implied a strong positive correlation between this paired lncRNA and mRNA (Fig. 6B, correlation coefficient = 0.795, *p* < 0.0001). Moreover, to explore the possible role of *TMEM220* in HCC, we retrieved expression profile of this gene from several databases. From Fig. 6C, we can see a liver-specific expression of *TMEM220* among different types of normal tissue. However, this tissue-specific expression pattern disappeared in liver cancer cell when compared with multiple tissue-derived cancer cell lines.

## Discussion

Nowadays, much is known about the cellular changes and etiological agents responsible for HCC, while the underlying molecular pathogenesis of HCC is still unclear. Although much progress has been made, many issues still remain unresolved, such as the prognosis evaluation of HCC cases (Llovet, Burroughs & Bruix, 2003). Traditional prognostic staging models, including BCLC, Okuda and CLIP scoring systems, have been used to stage HCC (Bruix & Sherman, 2011; Grieco et al., 2005). Mounts of novel prognostic biomarkers based on gene expression also have been carried out to predict HCC prognosis (Budhu et al., 2013; Govaere et al., 2014; Hoshida et al., 2013). However, neither of which can distinguish every situation. Up to now, mounts of reports have regarded lncRNAs as oncogenes or tumor suppressors. Previous studies on lncRNAs in HCC mainly focused on well-known cancer related transcripts. For example, overexpression of *HOTAIR* and *MALAT-1* were found to be associated with tumor recurrence of HCC after liver transplantation (Lai et al., 2012; Yang et al., 2011). In addition, suppression of these two lncRNAs in HepG2 (liver cancer cell line) could reduce cell viability, invasiveness, and increase the sensitivity to apoptosis.

In the present study, we found 1,074 dysregulated lncRNAs in LIHC, some of which have been recognized to be associated with cancer. For example, *LINC01419* was reported to be significantly overexpressed in HBV-related and HCV-related HCC (Zhang et al., 2015), which was consistent with our result. A single nucleotide polymorphism lies in *HAGLR* was identified as a risk site for ovarian cancer (Burghaus et al., 2017). Expression of *HOTTIP* is associated with HCC patients’ clinical progression and survival outcome (Quagliata et al., 2014). *LINC01093* and *FAM99A*, both specific expression in liver tissue, was significantly down-regulated in HCC compared with normal livers, as well as lower in cirrhotic tissues relative to normal tissues (Degli Esposti et al., 2016). *LINC00844* expression was also down-regulated in HCC and breast cancer (Fu et al., 2015; Jin et al., 2015).

Through univariate and multivariate survival analysis, four lncRNAs were identified to have the ability to predict patients’ overall survival independently. We then developed a risk score model based on expression of the four prognostic lncRNAs. With this risk model, patients were divided into LRG or HRG, which exhibited strong predictive power for patients’ outcome. Patients in LRG are more likely to survival longer than those in HRG. Moreover, prediction power could be enhanced when fitted lncRNA in the model based on mRNA signatures, which also indicate the significance of lncRNA as a valuable supplement to the regular mRNA markers. In addition, other prognostic factors for HCC, like HBV infection status and pathological stage, also showed significant correlation with survival expectations in our multivariate Cox regression analysis. Therefore, we performed stratification analysis for above clinical factors; the results suggested the ability of our risk score model to distinguish patients with significantly different prognostic outcomes (except for patients without HBV infection subgroup).

To confirm our results, we first explored the expression profiles of above lncRNAs in other datasets, which are similar with our findings. We then search some databases to find appropriate cohort to make a cross-validation of our survival prediction model. Considering the fact that many microarrays are not designed for lncRNA, we didn’t find suitable dataset with both expression profiles of the four lncRNAs and detailed clinical data for survival analysis in GEO database. In Fujimoto’s et al. (2016) study, RNAseq analysis were performed on 254 liver tumors, which may be helpful for our validation. However, the original sequencing data deposited in European Gnome-phenome Archive (EGA) database were not publicly accessible, and the processed expression matrix were also not supplied by the author. As a result, we only make a partial cross-validation of the expression profiles of these four prognostic lncRNAs.

As the risk score model was constructed on the basis of lncRNAs expression value and its powerful discrimination of expected survival, differentially expressed mRNAs between LRG and HRG were considered to share similar expression patterns with these signature lncRNAs. On the other hand, these differentially expressed mRNAs can also reflect the possible cause of the significantly different prognosis between these two groups. Enrichment analysis revealed that up-regulated genes are mainly involved in cell cycle pathway and associated with multiple cancers. Those down-regulated genes, linked with a variety of metabolic or cardiovascular diseases, are mainly enriched in drug metabolism pathway, which are consistent with the poor prognosis in HRG.

Although accumulating studies have demonstrated that lncRNAs involve in diverse cancers, the underlying mechanisms are still not well understood. In Panzitt’s et al. (2007) study, lncRNA *HULC*, acting as a post-transcriptional regulator of gene expression, was detected to be highly specific up-regulation in HCC. Moreover, subsequent research has shown that HULC can trigger protective autophagy and attenuate the sensitivity of HCC cells to chemotherapeutic agents (Xiong et al., 2017). Among the four prognostic lncRNAs, *RP11-150O12.3* was recognized as an independent predictor of gastric cancer (GC) prognosis (Ren et al., 2016), which also related to survival time of patients with colorectal adenocarcinoma (Zeng et al., 2017). *CTC-297N7.9*, locating upstream of protein coding gene *TMEM220*, showed its protective role in tumorigenesis and progression. As a transmembrane protein, *TMEM220* is specifically highly expressed in liver tissue compared with other normal tissues. Recently, a study find that *TMEM220* was down-regulated and highly methylated in GC tissues compared to normal tissues. The demethylation experiment also implied that the methylation of *TMEM220* was inversely correlated with its expression (Choi et al., 2017). Therefore, we suggest that *CTC-297N7.9* may be able to regulate the methylation of *TMEM220*, or be involved in cell autophagy through the interaction with functional proteins, and then affect the prognosis of HCC patients.

## Conclusions

In summary, we recognized four lncRNAs associated with OS of HCC patients, and constructed a risk score model based on lncRNAs expression profiles to make a survival prediction. The four lncRNAs could independently predict OS both in univariate and multivariate survival analysis. Our risk score model had a strong power to distinguish patients with different survival outcomes. These results shown the possibility of lncRNAs as novel biomarkers and therapy targets for HCC. Moreover, this study also revealed the possible role of lncRNAs in occurrence and progression of liver cancer.

##  Supplemental Information

10.7717/peerj.3575/supp-1 Figure S1 Survival analysis of four independent prognostic lncRNA markers Kaplan-Meier survival curve for (A) *RP11-322E11.5* (B) *RP11-150O12.3* (C) *AC093609.1* and (D) *CTC-297N7.9.* Patients were divided into high or low expression group with median expression value as the cutoff.

10.7717/peerj.3575/supp-2 Figure S2 GO enrichment analysis of mRNAs deregulated in high risk group compared with low risk group (A) GO analysis of upregulated mRNAs. (B) GO analysis of downregulated mRNAs.

10.7717/peerj.3575/supp-3 Table S1 Comparison between lncRNAs, mRNAs and combined transcripts based risk score model in the overall and stratification survival analysis

10.7717/peerj.3575/supp-4 Table S2 The corresponding probe name of four lncRNAs among different microarray platforms

## Abbreviations

HCC: Hepatocellular carcinoma
ROC: Receiver operating characteristic curve
HBV: Hepatitis B virus
HCV: Hepatitis C virus
lncRNA: Long non-coding RNA
TCGA: The Cancer Genome Atlas
LIHC: Liver hepatocellular carcinoma
TANRIC: The Atlas of Noncoding RNAs in Cancer
GO: Gene ontology
KEGG: Kyoto Encyclopedia of Genes and Genomes
DO: Disease ontology
AUC: Area under the ROC curve
GTEx: Genotype-Tissue Expression
CCLE: Cancer Cell Line Encyclopedia
OS: Overall survival
HRG: High risk group
LRG: Low risk group
BCLC: Barcelona Clinic Liver Cancer
CLIP: The Cancer of the Liver Italian Program
GC: Gastric cancer

## Additional Information and Declarations

### Data Availability

The following information was supplied regarding data availability: The origin RNAseq data used in our study were all downloaded from the TCGA data portal: https://portal.gdc.cancer.gov/.
