# Supplementary figures and images for: Identification of four prognostic LncRNAs for survival prediction of patients with hepatocellular carcinoma
Source: PeerJ. 2017 Jul 18;5:e3575. doi: 10.7717/peerj.3575 (PMC5518732; doi:10.7717/peerj.3575)

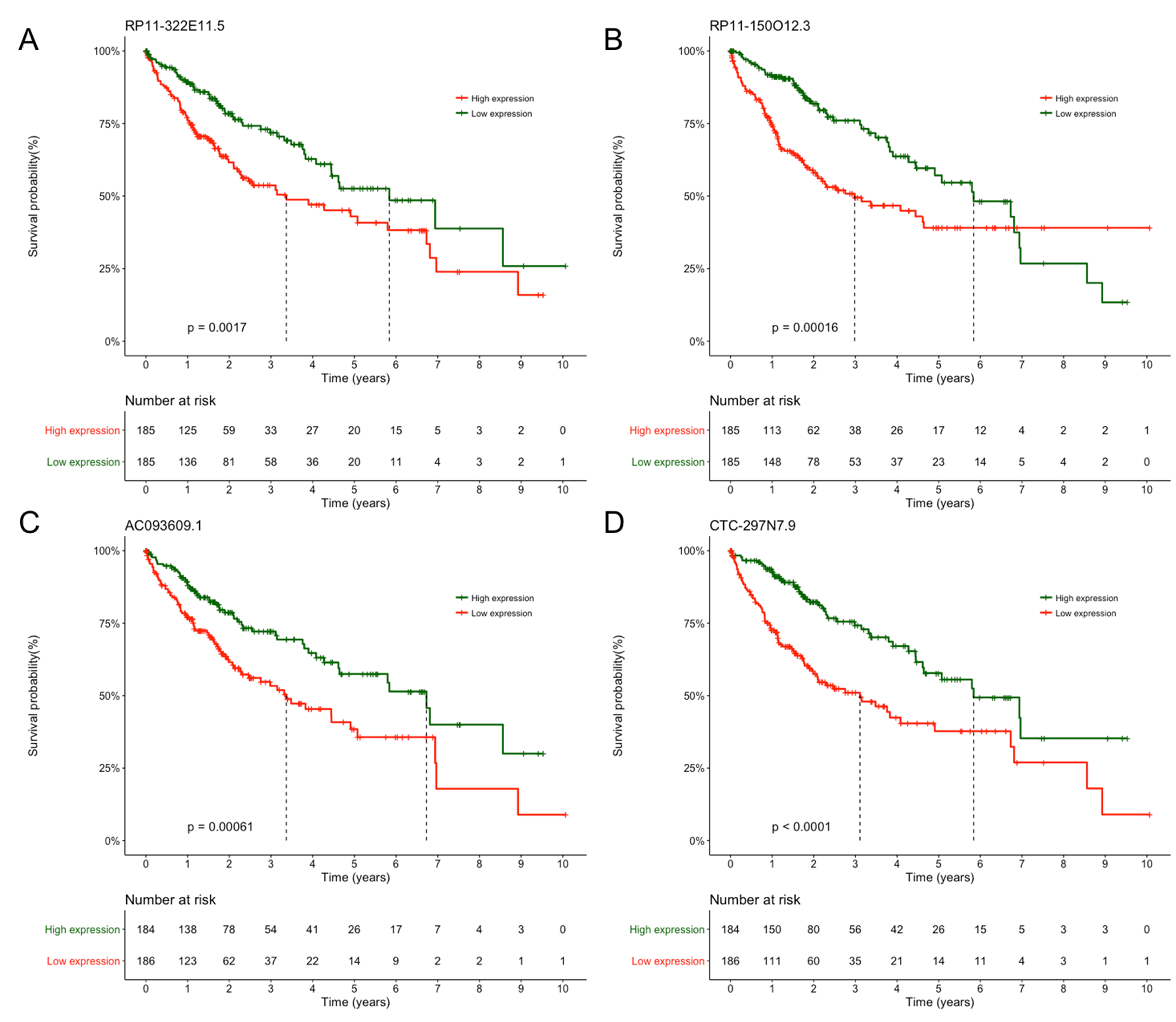

Supplement: Figure S1 — Kaplan-Meier survival curve for (A) RP11-322E11.5 (B) RP11-150O12.3 (C) AC093609.1 and (D) CTC-297N7.9. Patients were divided into high or low expression group with median expression value as the cutoff. [file peerj-05-3575-s001.png]

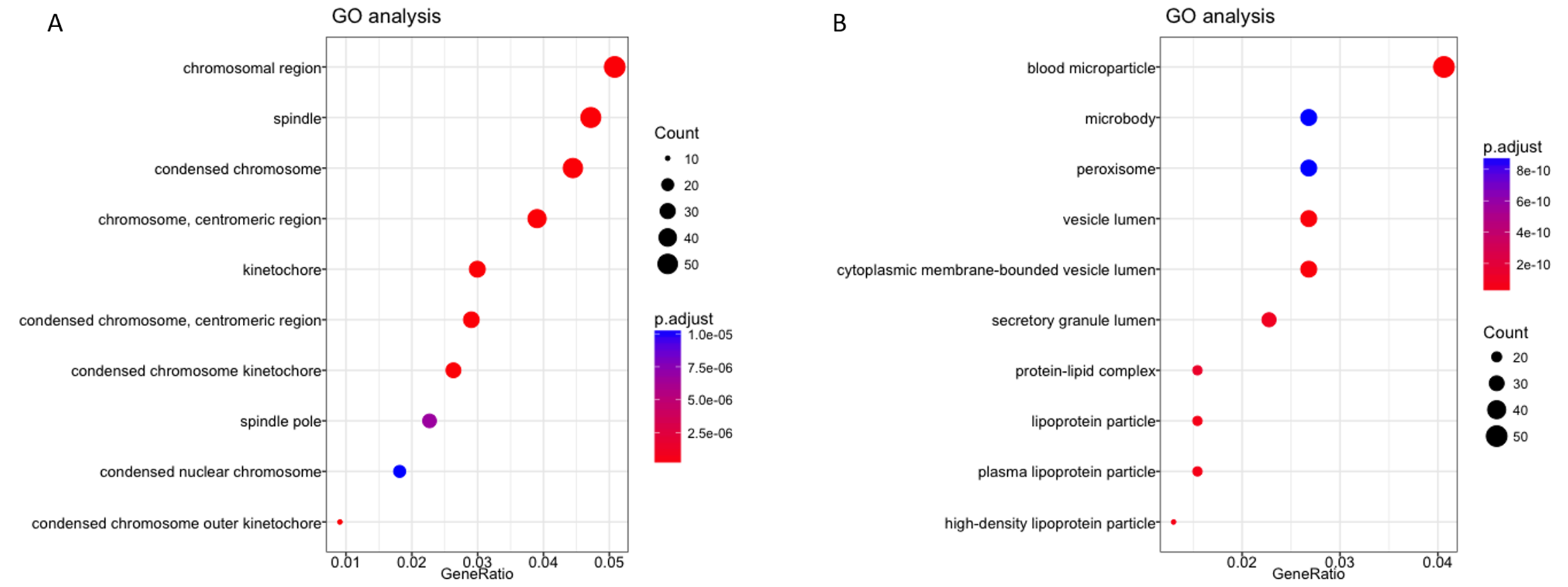

Supplement: Figure S2 — (A) GO analysis of upregulated mRNAs. (B) GO analysis of downregulated mRNAs. [file peerj-05-3575-s002.png]
