# Supplementary material for: Identification of four prognostic LncRNAs for survival prediction of patients with hepatocellular carcinoma
Source: PeerJ. 2017 Jul 18;5:e3575. doi: 10.7717/peerj.3575 (PMC5518732; doi:10.7717/peerj.3575)
Supplement: Table S1 [file peerj-05-3575-s003.docx]

**Table S1** Comparison between lncRNAs, mRNAs and combined transcripts based risk score model in the overall and stratification survival analysis.

|  | OS | HBV | No HBV | Stage i&ii | Stage iii&iv |
| --- | --- | --- | --- | --- | --- |
| lncRNA | 2.32E-14 | 4.30E-10 | 1.70E-04 | 5.20E-10 | 3.50E-04 |
| mRNA | 3.48E-13 | 1.90E-08 | 6.90E-05 | 1.40E-06 | 2.10E-05 |
| Combination | <2e-16 | 3.60E-11 | 6.30E-07 | 6.30E-11 | 3.20E-06 |

All the p values in the table were calculated with cox proportion hazard regression model, which treated risk score as independent variable.
