# Supplementary material for: Identification of four prognostic LncRNAs for survival prediction of patients with hepatocellular carcinoma
Source: PeerJ. 2017 Jul 18;5:e3575. doi: 10.7717/peerj.3575 (PMC5518732; doi:10.7717/peerj.3575)
Supplement: Table S2 [file peerj-05-3575-s004.docx]

**Table S2** The corresponding probe name of four lncRNAs among different microarray platforms.

|  | Microarray name | Probe name |
| --- | --- | --- |
| RP11-322E11.5 | Agilent-074348 | CUST_31542_PI430048170 |
| RP11-150O12.3 | HG-U133 Plus 2.0 | 232040_at |
|  | Agilent-028004 | A_33_P3802116 |
|  | Agilent-033010 | ASHG19A3A035685 |
|  | Agilent-052909 | A_33_P3802116 |
| AC093609.1 | HG-U133 Plus 2.0 | 241504_at |
|  | Agilent-039494 | A_19_P00320407 |
|  | Agilent-028004 | A_19_P00320407 |
|  | Agilent-033010 | ASHG19A3A013559 |
|  | Agilent-052909 | p29239 |
|  | Agilent-038314 | XLOC_002070 |
|  | Agilent-074348 | CUST_12815_PI430048170 |
| CTC-297N7.9 | Agilent-074348 | CUST_30019_PI430048170 |
|  | Agilent-052909 | p6678 |
